# Functional characterization of the trans-membrane domain interactions of the Sec61 protein translocation complex beta-subunit

**DOI:** 10.1186/1471-2121-10-76

**Published:** 2009-10-26

**Authors:** Xueqiang Zhao, Jussi Jäntti

**Affiliations:** 1 Research Program in Cell and Molecular Biology, Institute of Biotechnology, P.O. Box 56, 00014 University of Helsinki, Finland; 2 The State Key Laboratory of Plant Cell and Chromosomal Engineering, Institute of Genetics and Developmental Biology, Chinese Academy of Sciences, Beijing 100101, PR China

## Abstract

**Background:**

In eukaryotic cells co- and post-translational protein translocation is mediated by the trimeric Sec61 complex. Currently, the role of the Sec61 complex β-subunit in protein translocation is poorly understood. We have shown previously that in *Saccharomyces cerevisiae *the trans-membrane domain alone is sufficient for the function of the β-subunit Sbh1p in co-translational protein translocation. In addition, Sbh1p co-purifies not only with the protein translocation channel subunits Sec61p and Sss1p, but also with the reticulon family protein Rtn1p.

**Results:**

We used random mutagenesis to generate novel Sbh1p mutants in order to functionally map the Sbh1p trans-membrane domain. These mutants were analyzed for their interactions with Sec61p and how they support co-translational protein translocation. The distribution of mutations identifies one side of the Sbh1p trans-membrane domain α-helix that is involved in interactions with Sec61p and that is important for Sbh1p function in protein translocation. At the same time, these mutations do not affect Sbh1p interaction with Rtn1p. Furthermore we show that Sbh1p is found in protein complexes containing not only Rtn1p, but also the two other reticulon-like proteins Rtn2p and Yop1p.

**Conclusion:**

Our results identify functionally important amino acids in the Sbh1p trans-membrane domain. In addition, our results provide additional support for the involvement of Sec61β in processes unlinked to protein translocation.

## Background

The biosynthesis of secretory proteins requires translocation through the Sec61 channel into the endoplasmic reticulum (ER) [1]. The protein conducting channel is formed by three proteins and is conserved in evolution from archaea and bacteria to eukaryotic cells [2,3]. The largest subunit forming the actual protein conducting channel is named Sec61α in eukaryotes and SecY in bacteria and archaea. The β-subunit is termed Sec61β in metazoans and SecG in bacteria and archaea, whereas in yeast *Saccharomyces cerevisiae *(*S. cerevisiae*) two homologous β-subunits, Sbh1p and Sbh2p exist. The γ-subunit is called Sec61γ in mammalian cells, Sss1p in *S. cerevisiae *cells and SecE in bacteria and archaea. In *S. cerevisiae *two homologous trimeric Sec61 complexes exist. The Sec61 complex is composed of Sec61p, Sbh1p and Sss1p [4-7]. This complex together with Sec63p and Kar2p functions in co-translational translocation [8]. In association with Sec62p, Sec63p, Sec71p and Sec72p, Sec61 complex mediates also post-translational protein translocation as the so-called Sec complex [6,9]. In addition to its role in co- and post-translational translocation, the Sec61 complex has also been implicated in retro-translocation of misfolded proteins to the cytosol for degradation [10,11]. The second *S. cerevisiae *translocation complex, the Ssh1 complex, consists of the Sec61p homologue Ssh1p, the Sbh1p homologue Sbh2p, and Sss1p [2,8]. The Ssh1 complex has been shown to contribute to both co- and post-translational translocation and ER associated degradation (ERAD) [4,12].

Recent structural studies have shed light on the assembly and function of the α-subunit as the protein conducting channel [3,13,14]. In contrast, the functions of the β- and γ-subunits remain largely unclear. The β-subunit is not essential, but has a facilitating role in translocation in mammalian cells [15]. In addition to its interactions with Sec61p, the mammalian β-subunit has been shown to interact with signal peptidase [15] and the ribosome [16]. The yeast β-subunit, Sbh1p, was shown to act as a guanine nucleotide exchange factor for signal recognition particle receptor [17]. The two yeast β-subunits, Sbh1p and Sbh2p are encoded by non-essential genes. Deletion of either gene alone has no effect on growth, while the deletion of both *SBH1 *and *SBH2 *results in temperature-sensitivity for growth [4,7]. Different studies have reported variable effects of *SBH1 *and *SBH2 *deletion on protein translocation of the α mating pheromone precursor (ppαf), Kar2p, bacterial α-amylase and dipeptidyl aminopeptidase [4,15,18,19]. These results indicate a role for Sbh1p in co-translational protein translocation. Interestingly, the Sbh1p tm-domain alone is sufficient to support *in vivo *co-translational protein translocation and for interactions with Sec61 both in *S. cerevisiae *and in *S. pombe *[18-20].

The non-essential role of *SBH1 *and *SBH2*, in contrast to the essential genes encoding the α- and γ-subunits Sec61p and Sss1p, suggests that the β-subunits may have functions that are not directly linked to protein translocation. Previously, a stabilizing role in the translocation complex has been suggested for both β- and γ-subunits [21]. Both in mammalian and yeast cells, β-subunits have been shown to co-immunoprecipitate with the exocyst complex components Sec8p, Sec10p and Sec15p [22,23] whose main function is thought to be tethering of transport vesicles to the plasma membrane [24]. In addition, *SBH1 *over-expression has been shown to rescue the temperature-sensitive growth phenotype of several exocyst subunit mutations [22,23].

It appears that a pool of Sbh1p exists outside Sec61 complex and that these Sbh1p proteins can interact with Rtn1p, a member of the reticulon family proteins [18]. Rtn1p, together with Rtn2p and the DP1/Yop1p family protein Yop1p have been shown to function in modulation of the ER membrane structure [25-27]. Interestingly, in addition to its interactions with Sbh1p, Rtn1p has also been shown to co-purify with Exocyst subunit Sec6 [28]. Collectively, the current data suggests that Sbh1p displays complex molecular interactions and that its function may not be restricted to protein translocation. In this study we characterize the molecular determinants that are important for the function of the Sec61 protein translocation complex β-subunit tm-domain function. In addition, we show that this β-subunit interacts with ER-resident reticulon protein complexes.

## Methods

### Yeast strains

The yeast strains used are summarized in Table 1. Yeast cells were cultivated aerobically on plates or in shaker flasks at indicated temperatures essentially as described previously [29]. All strains used are congenic to H304 (NY179) (from P. Novick, Yale University). Gene deletions and carboxyl-terminal tagging of proteins were achieved with the PCR-based method using pFA6a-kanMX4, pFA6a-natNT2, pYM-hphNT1, pYM1 and pYM20 as templates ([30] from Michael Knop, EMBL, Heidelberg). G418 (Invitrogen) was used at a concentration of 200 μg/ml, nourseothricin at 100 μg/ml (Werner BioAgents, Germany), and hygromycin (EMD Biosciences, Inc.) at 300 μg/ml. Yeast transformations were carried out as described previously [31]. Complementation and suppression tests were performed as described previously [23].

**Table 1:** Yeast strains used in the study

**Strain**	**Genotype**	**Source/reference**
H304	*MAT***a ***ura3-52 leu2-3,112*	P. Novick (NY179)
H3232	*MAT***a***sbh1::kanMX4 sbh2::hphMX leu2-3,112 ura3-52*	[18]
H3429	*MAT***a***RTN1:3HA-kanMX4 sbh1::hphMX leu2-3,112 ura3-52*	[18]
H3431	*MAT***a***RTN1:RTN1-3HA-kanMX4, leu2-3, 112 ura3-52*	[18]
H3543	*MAT***a ***sbh1::kanMX4 sbh2::hphMX leu2-3,112 ura3-52 trp1::natNT2*	[18]
H3544	*MAT***a***RTN2:RTN2-3HA-kanMX4, leu2-3, 112 ura3-52*	This study
H3607	*MAT***a***RTN1:RTN1-3HA-kanMX4 YOP1:YOP1-9myc-hphMX leu2-3,112 ura3-52*	This study
H3609	*MAT***a***RTN1:RTN1-3HA-kanMX4 RTN2:RTN2-9myc-hphMX leu2-3,112 ura3-52*	This study
H3722	*MAT***a***YOP1:YOP1-9myc-natNT RTN1:RTN1-3HA-kanMX sbh1::hphMX leu2-3,112 ura3-52*	This study
H3723	*MAT***a***RTN2:RTN2-9xmyc-natNT RTN1:RTN1-3HA-kanMX sbh1::hphMX leu2-3,112 ura3-52*	This study

### SBH1 mutagenesis and library construction

Random mutagenesis for Sbh1p tm-domain encoding DNA segment was carried out by error-prone PCR using Goldstar DNA Polymerase (Eurogentec). PCR was performed in a 50 μl volume (30 cycles of 30 s at 94°C, 30 s at 56°C, and 1 min at 72°C), using 0.1 μg of template plasmid (YEpSBH1(50-75) [18] (Table 2), 100 pmol of each primer (5'-GCATTTCTAGAATGCTAAGAGTAGATCCCTTAG-3' and 5'-GCATTCTCGAGTTAAGAAATAACATGTAATGC-3'), 3 mM MgCl_2_, 0.5 mM MnCl_2_, three dNTPs at 0.12 mM, and the fourth at 0.016 mM and 2 units of Goldstar polymerase. Amplification products were gel-purified, pooled, digested with XbaI and Xhol restrictions enzymes and ligated into identically cut pVT102U [32]. Approximately 10,000 colonies were obtained, scraped into medium and grown overnight at 37°C followed by isolation of the plasmid DNA. After transformation of strain H3232 with this library, approximately 300 transformants were picked and regrown overnight at 30°C as patches on SC-Ura plates. These plates were then replicated to 30°C or 38.5°C and their growth was followed for 3 days. Site-directed mutagenesis was performed by QuickChange protocol (Stratagene) using appropriate mutagenic primers and Phusion DNA polymerase (Finnzymes). All constructs were verified by sequencing.

**Table 2:** Yeast expression vectors used in this study

**Plasmid name**	**Insert**	**Marker**	**Source**
pVT102U	-	*URA3*	[32]
YEpSBH1	SBH1(1-82)	*URA3*	This study
YEpSBH1-TM	SBH1(50-75)	*URA3*	This study
YEpBIO-(FL)SBH1	BIO-SBH1(1-82)	*URA3*	This study
YEpBIO-SBH1-TM	BIO-SBH1(50-75)	*URA3*	This study
YEpBIO-SBH1FL(DM)	BIO-SBH1(1-82, P54S V57G)	*URA3*	This study
YEpBIO-SBH1TM (DM)	BIO-SBH1(50-75, P54S V57G)	*URA3*	This study
YEpBIO-SBH1TM(SM1)	BIO-SBH1(50-75, P54S)	*URA3*	This study
YEpBIO-SBH1TM(SM2)	BIO-SBH1(50-75, V57G)	*URA3*	This study
YEpSBH1-FL(DM)L	BIO-SBH1(1-82, P54S V57G)	*LEU2*	This study
YEpSBH1-TM(DM)L	BIO-SBH1(50-75, P54S V57G)	*LEU2*	This study
YEpSBH1-TM(SM1)L	BIO-SBH1(50-75, P54S)	*LEU2*	This study
YEpSBH1-TM(SM2)L	BIO-SBH1(50-75, V57G)	*LEU2*	This study
p425ADH	-	*LEU2*	[35]
p426ADH	-	*URA3*	[35]
YEpBIO-SBH1TM-M1	BIO-SBH1(50-75, I65T F66S V73A I74T)	*URA3*	This study
YEpBIO-SBH1TM-M2	BIO-SBH1(50-75, A70S)	*URA3*	This study
YEpBIO-SBH1TM-M3	BIO-SBH1(50-75, F59L V62A F66L V69A L71S I74T)	*URA3*	This study
YEpBIO-SBH1TM-M4	BIO-SBH1(50-75, L60P)	*URA3*	This study
YEpBIO-SBH1TM-M5	BIO-SBH1(50-75, F64S S67P H72R)	*URA3*	This study
YEpBIO-SBH1TM-M6	BIO-SBH1(50-75, I65V S67P)	*URA3*	This study
YEpBIO-SBH1TM-M7	BIO-SBH1(50-75, V57A I65N L71P)	*URA3*	This study
YEpBIO-SBH1TM-M8	BIO-SBH1(50-75, V57E L60P F66S)	*URA3*	This study

All plasmids are 2 μ-type and contain the *ADH1 *promoter

### Plasmids construction

Standard DNA methods were used. Oligonucleotides were purchased from Sigma Genosys or Invitrogen. The plasmids used for gene expression in yeast are listed in Table 2. YEpSBH1(1-82) (YEpSBH1) and YEpSBH1(50-75) (YEpSBH1-TM) have been described previously [18]. *SBH1 *containing BIO-tag was constructed by amplifying with BamHI/XhoI containing oligonucleotides a fragment encoding the biotin acceptor peptide (BIO-tag) from *Propionibacterium shermanii *transcarboxylase [33]. This fragment was added in front of *SBH1 *mutants (obtained from the library screen) by nested PCR using pFA6a-HBH-kanMX46 ([34] from Peter Kaiser, University of California) as the template for BIO-tag. Generated fragments were cloned into p426ADH plasmid [35]. All constructs generated by PCR were sequenced.

### Antibodies

The Sec61p and Sbh1p antibodies have been described previously [18]. Monoclonal anti-HA antibody (12CA5) was purchased from Roche Applied Science. Monoclonal anti-myc (9E10) antibody was obtained from Kristiina Takkinen (VTT Technical Research Centre of Finland). Antibody dilutions used in Western blotting were 1:2000 for anti-HA, 1:1000 for anti-myc, anti-Sbh1p, 1:4000 and 1:2000 for anti-Sec61p. HRP-conjugated goat anti-mouse and anti-rabbit antibodies (Bio-Rad) were diluted 1:2000.

### Pull-downs and immunoprecipitations

Membrane protein-enriched fractions were isolated as described [18] and used for pull-down of BIO-tagged or immunoprecipitation of HA-tagged proteins. Streptavidin or anti-HA (12CA5) antibody-coupled Dynabeads (Dynal) were used for precipitation of BIO-tagged Sbh1p proteins or Rtn1p-3HA and Rtn2p-3HA. Coupling of anti-HA antibodies to Protein G-coupled magnetic beads was carried out according to manufacturer's instructions (Dynal). Incubation with the lysate was carried out at 4°C by end over end rotation for 1 h followed by 5 washes at 4°C by rotation, 15 min each. The bound proteins were eluted twice with 0.1% SDS treatment for 5 min at 95°C. The eluates were adjusted to 100 mM DTT, heated as described above, and subjected to SDS-PAGE and Western blotting. The protein bands were detected with specific antibodies or HRP-conjugated streptavidin (Molecular Probes) diluted 1:5000 and visualized with the ECL detection system (Amersham Biosciences).

### Ubiquitin-assisted Translocation Assay (UTA)

Plasmids YEpSBH1-FL(DM)L (full length *SBH1 *with mutations P54S V57G), YEpSBH1-TM(DM)L (tm-domain encoding *SBH1 *with mutations P54S V57G), YEpSBH1-TM(SM1)L (tm-domain encoding *SBH1 *with mutation P54S) and YEpSBH1-TM(SM2)L (tm-domain encoding *SBH1 *with mutation V57G) were transformed into H3543 strain containing plasmids encoding either Suc2_23_, Sec2_277_, Dap2_300 _(from N. Johnsson, University of Muenster, Germany, and J. Brown, Newcastle University, United Kingdom), or the empty vector pRS314 [1,36]. Patches of transformants grown on SC-Trp-Leu were replicated on SC-Trp-Leu-Ura plates and their growth was followed for 3 days at 24°C.

## Results

### Characterization of Sbh1p trans-membrane domain mutants

We have previously shown that the Sbh1p tm-domain is capable of interacting with the Sec61p-Sss1p complex and sufficient to rescue the co-translational translocation defect in *sbh1Δ sbh2Δ *cells [18]. In order to identify regions in the Sbh1p tm-domain that are important for its function we generated, by error-prone PCR, a DNA library containing random mutations in the tm-domain encoding fragment. This library was transformed into the temperature-sensitive *sbh1Δ sbh2Δ *cells, approximately 300 transformants were picked, and their capability to grow at the restrictive temperature was monitored. Approximately 30% of the transformants could not grow at 38.5°C, indicating that the Sbh1p tm-domain had acquired mutations in functionally important amino acids. The plasmids from 60 non-functional and functional clones were subjected to sequencing. This revealed that most of the plasmids (93%) contained mutations that resulted in one or more amino acid changes within the Sbh1p tm-domain. The *sbh1 *mutants, which could not rescue temperature-sensitivity in *sbh1Δ sbh2Δ *cells, contained either an early stop codon or more than one amino acid changes. A selection of mutants is shown in Table 3. The results demonstrate that the Sbh1p tm-domain tolerates a surprising number of non-conservative amino acid changes without a significant effect on *sbh1Δ sbh2Δ *cell growth.

**Table 3:** Mutants analyzed in this study

**Mutant/WT**	**Sequence**	**Rescue**
SBH1P TM	MLRVDPLVVLFLAVGFIFSVVALHVIS	Yes
SBH1P TM SP1	MLRVD**S**LVVLFLAVGFIFSVVALHVIS	Yes
SBH1P TM SP2	MLRVDPLV**G**LFLAVGFIFSVVALHVIS	Yes
SBH1P TM DP	MLRVD**S**LV**G**LFLAVGFIFSVVALHVIS	No
SBH1P TM mutant 1	MLRVDPLVVLFLAVGF**TS**SVVALH**AT**S	Yes
SBH1P TM mutant 2	MLRVDPLVVLFLAVGFIFSVV**S**LHVIS	Yes
SBH1P TM mutant 3	MLRVDPLVVL**L**LA**A**GFI**L**SV**A**A**S**HV**T**S	Yes
SBH1P TM mutant 4	MLRVDPLVVLF**P**AVGFIFSVVALHVIS	Yes
SBH1P TM mutant 5	MLRVDPLVVLFLAVG**S**IF**P**VVAL**R**VIS	No
SBH1P TM mutant 6	MLRVDPLVVL**S**LAVGF**V**F**P**VVALHVIS	No
SBH1P TM mutant 7	MLRVDPLV**A**LFLAVGF**N**FSVVA**P**HVIS	No
SBH1P TM mutant 8	MLRVDPLV**E**LF**P**AVGFI**S**SVVALHVIS	No
SBH1P TM mutant 9	ML**T**VDPLVVLFLAVGFI**L**SVVALHVIS	Yes
SBH1P TM mutant 10	MLRVDPLV**G**L**L**LAVGFI**L**SVVALHVIS	Yes
SBH1P TM mutant 11	MLRVDPLVVLFLAVGFI**P**SV**A**ALHVIS	Yes
SBH1P TM mutant 12	MLRVDPL**A**V**S**FLA**A**GFI**P**SVVALHVIS	Yes
SBH1P TM mutant 13	MLRVDPL**A**VLF**P**AVGFI**Y**SVVALHVIS	Yes
SBH1P TM mutant 14	MLRVDPLVVLFLAVGF**T**FSVVALHVIS	Yes
SBH1P TM mutant 15	MLRVDP**S**VVLFLAVG**L**IFSVVAL**R**VIS	Yes
SBH1P TM mutant 16	M**PS**VDPLVVLF**P**AVGFIFSVVALHVIS	Yes
SBH1P TM mutant 17	MLRVDPLVVLFLAVGF**V**FSVVALHVIS	Yes
SBH1P TM mutant 18	MLRV**G**PLVVLFLAVGFIFSVVALHVIS	Yes
SBH1P TM mutant 19	MLRVDPLVVLFLAVG**S**IFSVVALHVIS	Yes
SBH1P TM mutant 20	MLRVDPLVVLFLAVGFIF**P**VVALHVIS	Yes
SBH1P TM mutant 21	MLRV**G**PLVVLFLAVG**L**IFSV**A**AL**R**VIS	No
SBH1P TM mutant 22	M**P**RVDPLVVL**S**LAVGF**V**F**P**VVALHVIS	No
SBH1P TM mutant 23	MLR**A**DPLV**A**LFLAVG**S**IFSVVALHVIS	No
SBH1P TM mutant 24	M**P**RVDPLV**E**LF**P**AVGFI**S**SVVALHVIS	No
SBH1P TM mutant 25	M**P**RVDPLV**A**LFLAVGF**N**FSVVA**P**HVIS	No
SBH1P TM mutant 26	MLRVDPLV**A**LFLAVG**LT**FSVVA**Y**RYFL	No
SBH1P TM mutant 27	MLRVDPL**AAS**F**P**AVG**SYLP**V**A**ALHVIS	No
SBH1P TM mutant 28	MLRVDPLV**ASS**LAVG**LV**F**P**VVALHVIS	No

**Black **color highlights the mutated amino acid

**Figure 1:**
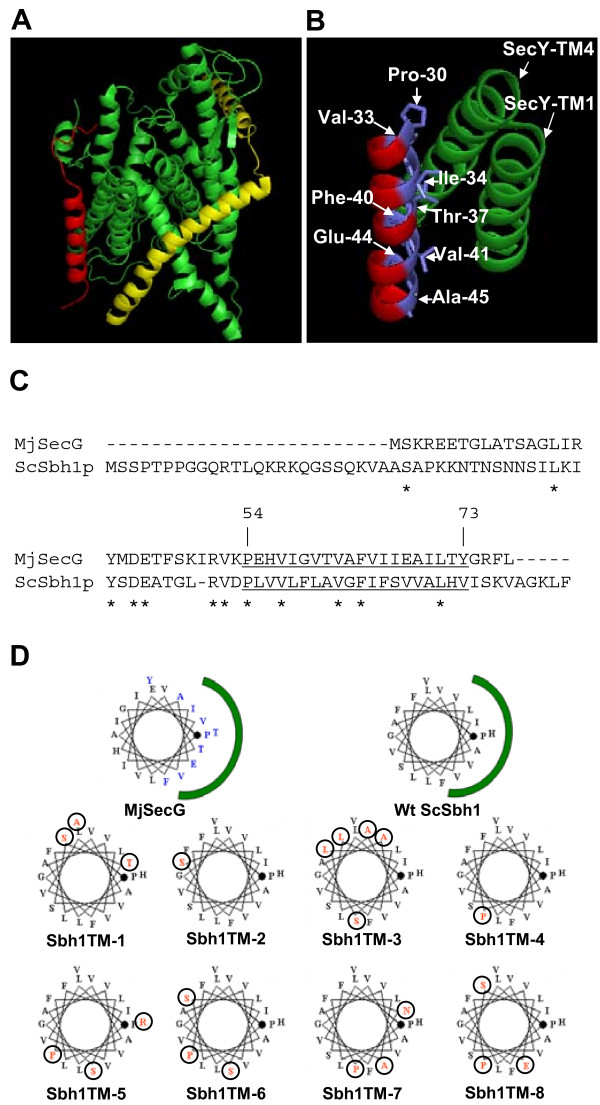
**Mutations in the Sbh1p trans-membrane domain**. A, The structure of *Methanococcus jannaschii *SecYEG complex. Green, SecY, yellow, SecE and red SecG. B, a view at the interface of SecG-SecY in *M. jannaschii*. Amino acids with blue are to face SecY complex according to crystal structure of SecY in *M. jannaschii*. Pictures were generated using PyMol (v0.99rc6). C, Sequence comparison of *M. jannaschii *SecG and *S. cerevisiae *Sbh1p. Asterisks indicate identical amino acids. The predicted tm-domain is underlined. The numbering is based in Sbh1p sequence. D, a helical wheel diagram of the predicted trans-membrane domains of *M. jannaschii *SecG (top left), wt Sbh1p (top right) and a selection of eight mutants identified (Sbh1TM-1 to Sbh1TM-8). The green arch indicates the side of β-subunit that is facing the SecG and proposed to face Sec61p. MjSecG amino acids coloured blue face MjSecY (top left wheel). Mutated amino acids in Sbh1TM1-8 are highlighted with a circle and red color. Proline (P) marked with the black dot corresponds to P54 in ScSbh1p and P30 in MjSecG. From this point the wheel is read anti-clockwise.

Recent studies have revealed high resolution SecY-complex structures from different species [3,13,14]. While no high resolution structure of the eukaryotic Sec61 translocation channel exists, a lower resolution structure of the mammalian non-translating ribosome-bound Sec61 complex was recently obtained [37]. This structure, however, does not reveal details of the β-subunit. The Sec61 β homologue SecG is the least conserved subunit among SecY complexes. In some species such as *Thermotoga maritima *SecG is comprised of two trans-membrane helices, whereas the structure of *Methanococcus jannaschii *trimeric SecY translocation complex reveals that the β-subunit traverses the membrane with a single α-helix with one side of the helix interacting with the α-subunit of the complex (Figure 1A and 1B, [3]).

**Figure 2:**
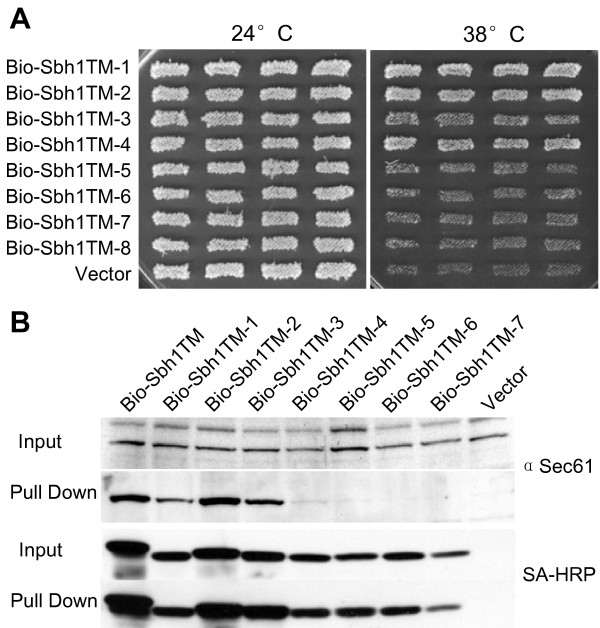
**Functional characterization of the Sbh1p tm-domain mutants**. A, Multicopy suppression of temperature-sensitivity of *sbh1Δ sbh2Δ *cells (H3232) by different mutant forms of *SBH1 *tm-domain. The *sbh1Δ sbh2Δ *cells were transformed with BIO-tagged *SBH1 *tm-domain mutants expressed from the *ADH1 *promoter in p426ADH and the growth of four independent transformants was monitored at 38 and 24°C. B, Lysates prepared from *sbh1Δ *cells (H3429) expressing BIO-tagged Sbh1p TM, mutants TM-1 to TM-7 or the empty vector were subjected to pull-down with streptavidin-coated magnetic beads. Beads and input samples were analyzed by Western blotting with anti-Sec61 antibodies or with HRP conjugated streptavidin to detect different versions of BIO-Sbh1p.

In order to understand where the obtained mutations are located in the Sbh1p tm-domain structure, we made use of the similarity of SecG tm-domains from different species and deduced a model for Sbh1p association with Sec61α (Figure 1C and 1D). The tm-domains of Sbh1p homologues have a highly conserved proline residue at the membrane/cytosol boundary complex [3]. We used this residue to align the Sbh1p tm-domain with the *Methanococcus jannaschii *SecG tm-domain (Figure 1C). The *M. jannaschii *SecG tm-domain has one side of its tm domain facing the tm-domains 1 and 4 of SecY (Figure 1B). To test the effect of mutations in different locations of the Sbh1p tm-domain α-helix, eight mutants were selected for further analysis (Figure 1C). Based on the model, mutations in TM-1, TM-2, TM-3 and TM-4 are mostly oriented away from the Sec61 whereas mutations in TM-5, TM-6, TM-7 and TM-8 are facing Sec61 (Figure 1C). When tested for their capability to rescue the temperature-sensitivity of the *sbh1Δ sbh2Δ *cells, mutants TM-5, TM-6, TM-7, and TM-8 were unable to rescue temperature-sensitivity of this strain. Mutant TM-3 was partially functional and mutants TM-1, TM-2 and TM-4 displayed normal growth (Figure 2A).

The Sbh1p tm-domain is sufficient for co-purification with the two other subunits of the Sec61 complex, Sec61p and Sss1p [18]. We next tested the effect of the selected mutations in the tm-domain for interaction with Sec61. As the tm-domain is only 25 amino acids long and not recognized by the antibodies available, for detection and pull-down experiments, we made use of the BIO-tag derived from *Propionibacterium shermanii *transcarboxylase that is biotinylated *in vivo *in yeast [33,34]. Lysates were prepared from *sbh1Δ *cells that expressed, from a plasmid, different mutant variants of Sbh1p tm-domain with a BIO-tag. These lysates were subjected to pull-down experiments with streptavidin-coupled magnetic beads followed by SDS-PAGE and Western blotting and detection with antibodies to Sec61p or streptavidin conjugated HRP (Figure 2B). The results show that the three mutants (mutants TM-5, TM-6 and TM-7) that could not rescue the temperature-sensitive phenotype of *sbh1Δ sbh2Δ *cells, lose their interaction with Sec61p. The TM-8 mutant protein levels were very low (data not shown) and were excluded from this analysis. At the same time mutant TM-2 co-purified Sec61 as efficiently as the wt Sbh1p tm-domain. Mutant TM-1 and TM-3 displayed reduced interaction with Sec61. Mutant TM-4 that could rescue temperature-sensitivity of *sbh1Δ sbh2Δ *cells could only weakly copurify Sec61 (Figure 2B).

### The double mutation P54S V57G inactivates the Sbh1p trans-membrane domain

**Figure 3:**
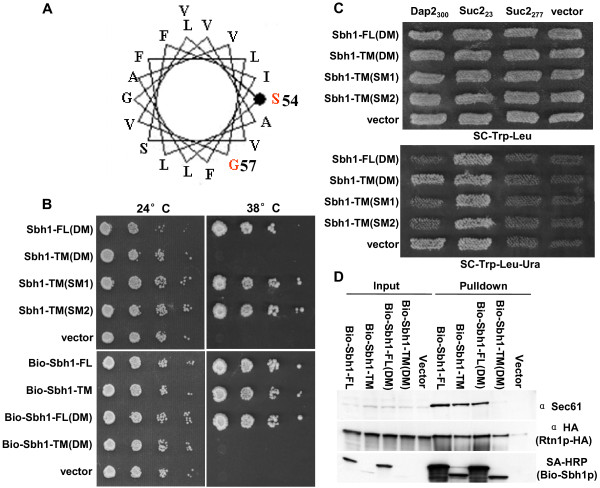
**The P54S V57G Sbh1p tm-domain double mutant is unable to rescue loss of Sbh1p and Sbh2p**. A, A wheel presentation of the P54S V57G Sbh1p tm-domain mutant. B, Growth of *sbh1Δ sbh2Δ *cells (H3232) transformed with plasmids encoding *SBH1 *tm-domain mutants with or without BIO-tag. C, UTA translocation assay on *sbh1Δ sbh2Δ *cells (H3543) transformed with plasmids encoding reporter proteins Suc2_23_, Sec2_277_, Dap2_300_, or the empty vector pRS314, or with plasmids encoding full length Sbh1p with P54S V57G mutations (Sbh1-FL(DM)), Sbh1p tm-domain with P54S (Sbh1-TM(SM1)), Sbh1p tm-domain with V57G (Sbh1-TM(SM2)) or Sbh1p tm-domain with P54S V57G (Sbh1-TM(DM)) or the empty plasmid. The growth of transformants was tested on SCD-Trp-Leu or on SCD-Trp-Leu-Ura plates to score for translocation of the Ura3p containing reporters. D, Western blot analysis with anti-HA (for Rtn1p-HA), Sec61p antibodies or with HRP conjugated streptavidin (for versions of BIO-Sbh1p) of pull-downs from *sbh1Δ *cells (H3429) expressing BIO-tagged Sbh1p(P54S V57G), Sbh1p TM(P54S), Sbh1p TM(V57G), Sbh1p TM(P54S V57G) or an empty plasmid p426ADH.

Sequence homology between tm-domains of MjSecG and Sbh1p and our mutant collection suggest that mutations D53G, P54S, V57G, F64S, S67P, and V69A could affect potentially important positions within the Sbh1p tm-domain. D53 is located at the cytosol/membrane boundary whereas P54, V57 and F64 are conserved between *M. jannaschii *and *S. cerevisiae*. However, the single mutations D53G, P54S, V57G, F64S, S67P and V69A did not inactivate the Sbh1p tm-domain as judged by the rescue of the temperature-sensitivity of *sbh1Δ sbh2Δ *cells (Table 3, Data not shown). Based on the data indicating that one side of the predicted Sbh1p tm-domain α-helix is especially sensitive for mutations, and on the sequence conservation within the Sbh1p homologues, we generated, by site directed mutagenesis, versions of Sbh1p that have P54S V57G mutations (Figure 3A). These mutations target the side of Sbh1p tm-domain α-helix that based on the SecY structure [3] would face Sec61p (Figure 1A and Figure 3A). When expressed in *sbh1Δ sbh2Δ *cells this double mutant (DM) was unable to rescue the temperature-sensitive growth phenotype at 38.5°C whereas the single mutants P54S (SM1) and V57G (SM2) were as efficient as the wild type *SBH1 *to rescue the temperature-sensitive growth of *sbh1Δ sbh2Δ *cells (Figure 3B). The same was true when the BIO-tagged versions of *SBH1 *were tested in this experimental setup (Figure 3B lower panel). Both untagged and BIO-tagged full length (FL) Sbh1p containing both P54S and V57G mutations in the tm-domain were able to rescue *sbh1Δ sbh2Δ *cell growth at the restrictive temperature. This suggests that the cytosolic domain of Sbh1p contributes to Sbh1p function or interaction with Sec61 enables sufficient *in vivo *functionality of Sbh1p that has these mutations in the tm-domain.

Sbh1p has previously been implicated in co-translational translocation ([18]). We therefore tested whether the P54S V57G double mutant is functional in protein translocation using the UTA assay [36,38]. The UTA assay measures coupling of translation to translocation by utilizing reporter proteins which contain a yeast signal sequence, a "spacer" peptide, ubiquitin and finally Ura3p. When translation is closely coupled to translocation, the reporter protein fusion with Ura3p is translocated to the lumen of the ER before the ubiquitin folds and is cleaved by cytosolic proteases. Such cells can not survive without uracil in the growth medium as Ura3p needs to be in the cytosol to rescue lack of uracil in the growth medium. In cases where there is a delay between translation and translocation, ubiquitin folds, gets cleaved and Ura3p is released in the cytosol enabling growth in the absence of uracil. Thus, if Sbh1 mutant proteins expressed in *sbh1Δ sbh2Δ *cells can not support translocation of the Ura3p reporter protein into the ER lumen the cells should be able to grow in the absence of uracil in the growth medium. This would indicate that this Sbh1p mutant is inactivated for protein translocation. To test this *sbh1Δ sbh2Δ leu2-3,112 ura3-52 trp1Δ *cells were transformed with reporter plasmids encoding invertase or dipeptidyl aminopeptidase B (Dap2) signal sequence and 23 (Suc2_23_), 277 (Suc2_277_) or 300 (Dap2_300_) first amino acids, respectively, before the ubiquitin moiety and Ura3p. The same cells were also transformed with plasmids encoding Sbh1p tm-domain with single P54S (TM-SM1) or V57G (TM-SM2) or the P54S V57G double mutation (TM-DM) and with a plasmid encoding full length Sbh1p with the P54S V57G double mutation (FL-DM) or the empty vector. Suc2p uses the post-translational and Dap2p the co-translational translocation pathway [36]. The *sbh1Δ sbh2Δ *cells expressing Suc2_23 _grew in all cases in the absence of uracil in the growth medium as the 23 amino acid linker enables rapid folding of the ubiqutin moiety, its cleavage and release of the Ura3p in the cytosol (Figure 3C, lower panel). The *sbh1Δ sbh2Δ *cells expressing Suc2_277 _did not grow in the absence of uracil indicating efficient post-translational translocation of this reporter protein in to the ER lumen in the absence of Sbh1 and Sbh2 proteins. Dap2_300 _reporter expressing *sbh1Δ sbh2Δ *cells also grew in the absence of uracil (vector, Figure 3C, lower panel) indicating a defect in co-translational translocation as previously reported [18,19]. The defect in co-translational translocation was rescued by reintroduction of full length Sbh1p FL-DM, Sbh1p TM SM1 or Sbh1p TM SM2, but not by expression of the Sbh1p tm-domain with the P54S V57G double mutation (TM-DM) (Figure 3C, lower panel). We conclude that wt Sbh1p, and the Sbh1p tm-domain with the single P54S or V57G mutation can rescue the co-translational translocation defect in *sbh1Δ sbh2Δ *cells whereas the Sbh1p tm-domain with the double mutation P54S V57G cannot.

### The Sbh1p P54S V57G trans-membrane domain double mutant does not bind to Sec61p

In order to test Sec61 binding of the Sbh1p tm-domain double mutant we made use of constructs which enabled expression of the point mutants with an amino-terminal BIO tag (Figure 3B, lower panel). These constructs or an empty vector were transformed into *sbh1Δ RTN1-3HA *cells, lysates were prepared and subjected for pull-downs with streptavidin coated beads. The pull-downs were analyzed by SDS-PAGE and Western blotting with antibodies against Sec61p, HA or with HRP-coupled streptavidin. The results show that although similar amounts of Sbh1TM-DM and Sbh1TM were pulled down with streptavidin coated beads, the trans-membrane domain with P54S V57G mutations interacted only weakly with Sec61 (Figure 3D). At the same time, Sec61p co-precipitated with full length Sbh1p, Sbh1p tm-domain and full length Sbh1p FL(DM). The P54S V57G mutations did not affect co-precipitation of Sbh1p with Rtn1p-HA (Figure 3D). These results support the growth rescue and protein translocation experiment results and suggests that P54 V57 mutations affect interaction of the Sbh1 tm-domain with Sec61p. When these mutations are present in the full length Sbh1p containing also the cytosolic domain, the interaction of Sbh1p with Sec61p is partially restored. This suggests the cytosolic domain contributes to the Sbh1p interaction with Sec61.

### The Sbh1p trans-membrane domain is sufficient for interaction with Rtn2p and Yop1p

**Figure 4:**
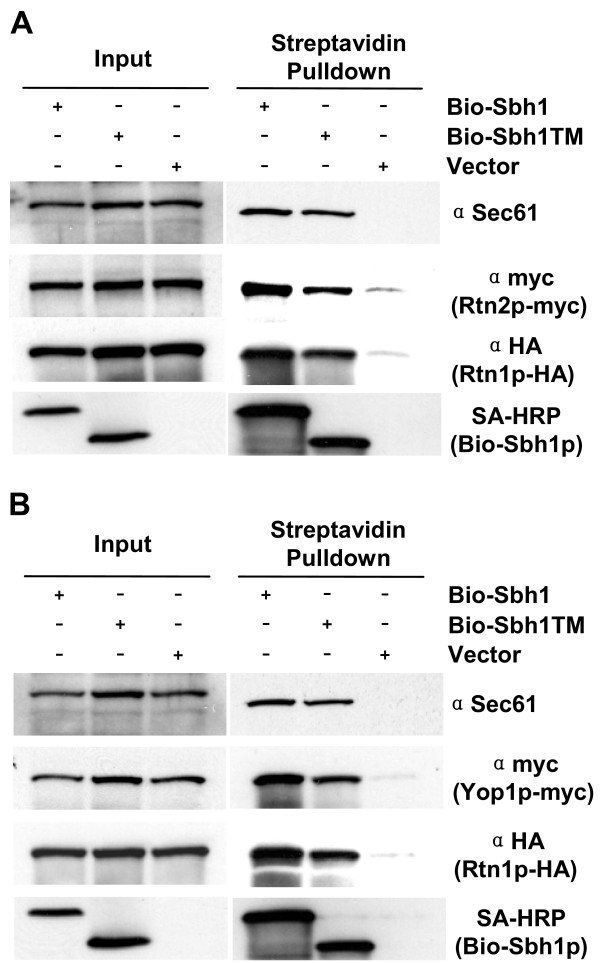
**Sbh1p co-immunoprecipitates with *S. cerevisiae *reticulon-homologues Rtn1p, Rtn2p and Yop1p**. A, The *sbh1Δ RTN1-3XHA RTN2-9xmyc *cells (H3723) or in B, *sbh1Δ RTN1-3XHA YOP1-9xmyc *cells (H3722) were transformed with YEpBIO-SBH1, YEpBIO-SBH1-TM, or an empty plasmid p426ADH. Lysates were prepared and subjected to pull-down with streptavidin-conjugated magnetic beads followed by SDS-PAGE and Western blotting. Proteins were detected with HRP-conjugated streptavidin and antibodies to Sec61p, myc and HA.

Our previous results together with the results above showed that Sbh1p is found in a complex with Rtn1p and that the tm-domain is sufficient for this interaction ([18], Figure 3D). In addition to Rtn1p, *S. cerevisiae *expresses two ER-localized membrane proteins, Rtn2p and Yop1p that are implicated in ER structure regulation [25-27]. In order to test whether also Rtn2p and Yop1p are found in protein complexes with Sbh1p, yeast strains were generated where *SBH1 *was deleted and *RTN1*, *RTN2 *or *YOP1 *were tagged either with HA or myc tags at their genomic locus where their expression was maintained under their endogenous promoters. These cells were transformed with an empty plasmid or a plasmid expressing a BIO-tagged version of either the full length Sbh1p or the Sbh1p tm-domain. Cells were subjected to pull-down experiments with streptavidin coated beads and the precipitated material was analyzed after SDS-PAGE and Western blotting with anti HA or myc antibodies. When Sbh1p full length or the tm-domain was pulled-down from lysates expressing BIO-Sbh1p, BIO-Sbh1p-tm, Rtn1p-HA, or Rtn2p-myc, both Rtn1p-HA and Rtn2p-myc were detected in the precipitates together with Sec61p. In cells harboring the empty vector instead of BIO-Sbh1p, only a weak signal for these proteins was detected (Figure 4A, vector). In lysates prepared from a strain expressing BIO-Sbh1p, BIO-Sbh1p-tm, Rtn1p-HA, or Yop1p-myc, both Rtn1p-HA and Yop1p-myc proteins co-purified with Sbh1p (Figure 4B). These results suggest that Sbh1p interacts with a larger protein complex that appears to contain all yeast reticulon-homology proteins.

## Discussion

We previously showed that the tm-domain of the *S. cerevisiae *Sec61 β-subunit Sbh1p is sufficient to ensure viability of *SBH1 SBH2 *deleted cells, support co-translational translocation and interact with the α-subunit Sec61p [18]. This indicates that the Sbh1p tm-domain possesses features that ensure specific functional interactions with the Sec61 protein translocation channel. Currently, no high resolution structure exists for the eukaryotic Sec61 complex and therefore it is not known how the β-subunit interacts with Sec61p. However, recently high resolution structures of the archaeal and prokaryotic SecY complexes have been resolved [3,13,14]. Furthermore, a lower resolution structure of the mammalian Sec61 complex bound to a non-translating ribosome was recently published [37]. This structure generated by electron microscopy is similar to the archaeal *M. jannashii *SecY complex structure indicating high overall structural conservation between species. Despite these significant advances, the detailed function of the protein translocation complex and especially the functions of the small SecG/β and SecE/γ subunits have remained elusive.

Among the protein translocation channel subunits the different SecG/β subunits are the least conserved by sequence making it difficult to define critical amino acids for their function. In order to gain a better understanding of the molecular determinants that are responsible for the *S. cerevisiae *Sec61 β-subunit tm-domain *in vivo *function, we set out to identify mutations in the Sbh1p tm-domain that could abolish its interaction with Sec61 and that would affect co-translational protein translocation. We found that the Sbh1p tm-domain tolerates significant changes in its amino acid composition with remarkably small effects on its function. When we used helical wheel projections to predict the distribution of these mutations, a pattern for the localization of inactivating mutations emerged. Using the archaeal *M. jannashii *SecY complex structure as a model, we observed that mutations on the side of Sbh1p tm-domain that in SecG would face SecY were especially prone to inactive Sbh1p function. This we confirmed by generating a version of Sbh1p tm-domain containing two mutations that localized in the putative Sbh1p-Sec61p interaction interface. Sbh1p tm-domains with these (P54S V57G) mutations bound only weakly to Sec61p and were unable to support co-translational translocation in the UTA protein translocation assay (Figure 3). The P54S mutation changes a proline that is predicted to be located at the cytosol/membrane boundary. This mutation changes the Sbh1p tm-domain to resemble its close homologue in *S. cerevisiae*, Sbh2p, which naturally possesses a serine at this position [4,7]. Sbh2p or its tm-domain alone have been shown to rescue the growth defect of *sbh1Δ sbh2Δ *cells [7,18-20,23]. This suggests that the V57G mutation is contributing significantly to inactivation of Sbh1p function and Sbh1p interaction with Sec61p. This conclusion is supported e.g. by mutations (V57A F64S) in the tm-domain mutant TM-23 (Table 3) which cause inactivation of Sbh1p. These mutations target the same side of the Sbh1p tm-domain alpha-helix like the inactivating mutations P54S and V57G. Both P54 and V57 are well conserved among SecG and Sec61 β homologues [3]. When P54S V57G mutations were generated in full length Sbh1p, co-translational translocation was rescued sufficiently for cell survival at elevated temperature and the interaction with Sec61p in immunoprecipitations was partially restored. It is therefore possible, that the cytosolic part of Sbh1p contributes to Sec61 binding or Sec61 protein functionality either directly or indirectly through association of Sec61p, the ribosome or other components involved.

The interactions displayed by the cytosolic domain are poorly understood as even in the solved high resolution structures of SecYEG complexes, these domains are poorly resolved [3,14]. One potential interaction partner could be the closely situated amino-terminal part of Sec61p [3,37]. Additional interaction partners could be provided by the reported binding of Sec61 complex subunits with the oligosaccharyl transferase complex [39]. Ultimately, a high resolution structure of the Sec61 complex is required to resolve the details of molecular interactions within this complex. However, our functional analysis of the Sbh1p tm-domain indicates the side of the Sec61 β-subunit tm-domain that is involved in Sec61p binding in *S. cerevisiae*. This suggests that the Sec61 β-subunit interaction with the Sec61 α-subunit is indeed well conserved in evolution. This conclusion is supported by the recent results showing that both the mammalian and *S. pombe *Sec61 β-subunits can complement for loss of Sbh1p and Sbh2p in *S. cerevisiae *[20].

We have shown previously that Sbh1p interacts with Rtn1p, a member of the reticulon family of proteins in yeast [18]. This interaction appears to take place between Sbh1p and Rtn1p outside the Sec61 complex [18]. Rtn1p and reticulon family proteins in general have been shown to play an important role in modulation of ER membrane tubulation both in *S. cerevisiae *and mammalian cells [25-27]. Additional components in this function in yeast are the second reticulon family protein Rtn2p and a member of the DP1/Yop1p family, Yop1p. Yop1 and reticulon proteins Rtn1 and Rtn2 have been shown to form complexes of reduced lateral mobility in the peripheral ER in *S. cerevisiae *[25,26]. Here we show that Sbh1p co-immunoprecipitates not only with Rtn1p, but also with Rtn2p and Yop1p. Our findings support the recent results showing that Rtn1p, Rtn2p and Yop1p form trimeric complexes [25-27]. The role of Sbh1p interaction with reticulon and Yop1 complexes is currently unclear. However, the mutations that affected Sbh1p interaction with Sec61p did not abolish interaction with Rtn1p.

We have previously shown that overexpression of *SBH1 *can specifically suppress the growth defect of several mutations in the exocyst complex [7,23]. In addition to these genetic interactions, Sbh1p also co-immunoprecipitates with exocyst subunits Sec15p and Sec8p in yeast cells [23]. Intriguingly, similar interactions and functional links were obtained in mammalian cells [22] indicating that the Sec61 β and exocyst complex interactions carry out a conserved function. Association of the exocyst complex with ER membrane components is further supported by data showing co-purification of the exocyst complex with Rtn1p [28]. The existence of a functional link between Rtn1p and the exocyst is also supported by the capability of *RTN1 *overexpression to suppress temperature-sensitive mutations in exocyst subunits Sec5p and Sec8p and induction of a cold sensitive phenotype in *sec15-1 *cells by *RTN1 *deletion (Zhao and Jantti, unpublished). Interestingly, recent results show a molecular interplay between Sec5p, TBK1 kinase, a preferentially ER-localized trans-membrane protein STING and Sec61 β in mammalian cells. STING and TBK1 are implicated in innate immunity regulation and it was proposed that STING and the protein translocation channel are functionally linked during innate immunity signaling [40]. The exocyst subunit Sec5p has also previously been shown to be involved in innate immunity through its interaction with TBK1 kinase and knock-down of the exocyst subunit Sec5p renderes cells defective for STING function [40,41]. These results together with our present data on Sec61 β-subunit interactions with reticulon/Yop1 protein complexes, suggest a complex molecular interplay between ER membrane proteins and the exocyst complex, possibly through interactions between the Sec61 β-subunit, the exocyst complex and reticulon proteins. The elucidation of these interactions and their role in the cell represents an interesting, yet demanding challenge for future studies.

## Conclusion

Our results support the existence of significant structural and functional conservation in Sec61 β/SecG subunits and show that Sec61 β in *S. cerevisiae *associates with reticulon protein complexes. Furthermore, these results suggest an interesting role for Sec61 β in cellular regulation in addition to its role in protein translocation.

## Authors' contributions

XZ and JJ planned and XZ performed the experiments. JJ drafted the manuscript. Both authors read, edited and approved the final manuscript.
